# Genetic and epigenetic studies of adiposity and cardiometabolic disease

**DOI:** 10.1186/s13073-017-0474-5

**Published:** 2017-09-18

**Authors:** Michael V. Holmes, Sara L. Pulit, Cecilia M. Lindgren

**Affiliations:** 1 Medical Research Council Population Health Research Unit at the University of Oxford, Oxford, UK; 2 Clinical Trial Service Unit & Epidemiological Studies Unit (CTSU), Nuffield Department of Population Health, University of Oxford, Oxford, UK; 3 Medical Research Council Integrative Epidemiology Unit, University of Bristol, Bristol, UK; 4 National Institute for Health Research, Oxford Biomedical Research Centre, Oxford University Hospital, Oxford, UK; 5 The Big Data Institute, Li Ka Shing Centre for Health Information and Discovery, University of Oxford, Oxford, UK; 6 Medical Population and Genetics Program, Broad Institute, Cambridge, MA USA; 7 Department of Genetics, University Medical Center Utrecht, Utrecht, The Netherlands; 8 Wellcome Trust Center for Human Genetics, Oxford University, Oxford, UK

## Abstract

Over 300 million adults are obese, but little is known about the impact of obesity on cardiovascular health. We discuss recent genetic and epigenetic studies of adiposity that indicate a causal role for general and central adiposity in cardiometabolic disease, and highlight potential mechanisms including insulin resistance and gene expression.

## Background

Obesity, an excess of adiposity in which the body mass index (BMI) is 30 kg/m^2^ or more, is a global public health crisis leading to increased prevalence of diabetes at an unprecedented scale and an associated increased risk of cardiovascular disease [1].

**Fig. 1:**
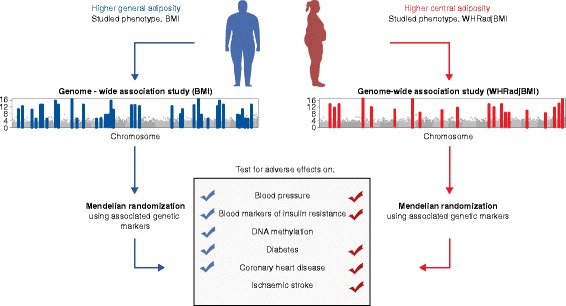
Relationships of general and central adiposity with cardiometabolic diseases and related traits identified through genetic and epigenetic studies. Two common phenotypes have been key to the genetic study of adiposity in humans: body mass index (*BMI*; in *blue*), which measures general adiposity; and waist-to-hip ratio adjusted for BMI *(WHRadjBMI*; in *red*), which captures central adiposity (that is, fat that collects around the central region of the body and may mark visceral fat deposits). Genome-wide association studies (*GWASs*) in BMI and WHRadjBMI have revealed 97 and 49 common variant loci, respectively, associated with the traits. While GWASs provide evidence for association between genetic variants and phenotypic outcomes, the variants implicated in these studies can be used in Mendelian randomization (*MR*) analyses to investigate causal relationships. MR studies using BMI-associated single nucleotide polymorphisms (SNPs) have established causal relationships of BMI on blood pressure, insulin resistance, DNA methylation (that is, alterations in gene expression), diabetes, and coronary heart disease. Similar studies, but for WHRadjBMI-associated SNPs, show similar causal relationships (excluding that for DNA methylation), and a causal role in stroke. The results indicate that not only general adiposity (indexed by BMI) but the distribution of adipose tissue in particular depots (indexed by WHRadjBMI) is crucial to the relationship between adiposity and cardiometabolic disease outcome

There is considerable inter-individual variation in how, where, and to what extent fat deposits around the body. For example, two individuals can have the exact same height and weight (that is, identical BMI, a crude measure of adiposity calculated by dividing weight by height squared) but have different cardiometabolic disease risk (Fig. 1) [1]. These differences may arise due to where fat is stored. For example, fat deposited around viscera (proxied by the measure of an individual’s waist-to-hip ratio (WHR)) may have different impacts on health compared to fat deposited subcutaneously or around the thighs [2]. Understanding the relationship between adiposity and disease and the mechanism(s) by which this relationship is mediated is critical if we are to find effective approaches to disease prevention.

## Associative and causal relationships

Observational studies provide strong evidence of positive associations between adiposity and cardiometabolic disease risk, but can suffer from bias and confounding. Randomized controlled trials (RCTs) are the gold standard for establishing causality. While the DIRECT trial provided reliable evidence for reduced type 2 diabetes (T2D) risk as a consequence of a lifestyle intervention leading to weight loss, only one RCT (LOOK-Ahead) has investigated the clinical impact of reduced caloric intake and increased physical activity on CVD risk, but this was stopped after 10 years due to a lack of efficacy. Recent studies of cardiometabolic disease have embraced an alternative approach: Mendelian randomization (MR), which exploits properties of the genome to make causal, rather than correlative, inferences on the relationship between an exposure and an outcome [3].

Initial MR studies in cardiometabolic disease focused on only a small number of variants associated with BMI or other adipose-related traits. Studies testing only a small number of single-nucleotide polymorphisms (SNPs) are potentially limited as BMI is a complex trait; a single locus is unlikely to provide a comprehensive proxy of a trait’s overall genetic architecture, which is probably comprised of hundreds of modest effect associations. Additionally, the identified locus may be pleiotropic with other traits [3]. Thus, early MR studies were at least in part hampered by inadequate numbers of genetic variants (limiting the proportion of explained BMI variance) and lack sufficient numbers of disease cases (limiting statistical power). Consequently, early MR studies yielded unreliable estimates, exemplified by an MR using 14 SNPs associated with BMI, coupled with a meta-analysis [4] of the available literature at the time; while showing robust associations with markers of inflammation, blood pressure, and diabetes, the study failed to identify the causal relationship between BMI and coronary heart disease (CHD) that more recent work [2] indicates is likely real.

The availability of large-scale genome-wide association study (GWAS) data generated from increasingly larger sample sizes [5, 6] has spurred additional methodological developments in MR. One such advance is two-sample MR, which exploits separate datasets for the SNP-to-exposure and SNP-to-outcome relationships, facilitating the inclusion of GWAS summary data into the analysis, and thus vastly increasing statistical power. A second advance has been the increase in the number of phenotype-associated SNPs, made possible by collaborative GWAS employing large samples and dense imputation reference panels (which facilitate the imputation of unobserved genotypes in the samples) [5, 6]. These developments in the field allowed, for example, two-sample MR analysis of 32 BMI SNPs and data from the Coronary Artery Disease Genome-wide Replication and Meta-analysis (CARDIoGRAM) plus the Coronary Artery Disease (C4D) Genetics consortium (CARDIoGRAMplusC4D) [7] to provide evidence that adiposity is causally implicated in the development of CHD. Notably, a one-sample MR analysis in the same study failed to detect the causal effect of BMI on incident CHD (highlighting the importance of adequate statistical power to obtain reliable estimates of effect).

Recent MR approaches are elucidating how distinct features of adiposity causally influence cardiometabolic disease risk through specific mechanisms. One study [8] used 32 genetic variants to investigate the effects of BMI on circulating blood-based metabolic markers, including inflammatory markers and a number of hormones including leptin and insulin. This demonstrates how GWAS data and MR can be integrated to implicate potential mediators of the relationship between BMI and cardiometabolic disease. The latest obesity GWAS, one examining BMI (which measures overall fat) and one looking at WHR adjusted for BMI (WHRadjBMI, which measures central adiposity), identified 97 and 49 common variants, respectively [5, 6]. Notably, these two GWAS reveal partially distinct genetic signatures in BMI and WHRadjBMI, prompting the question of whether central body fat has effects on cardiometabolic disease that are independent of total body fat. To address this question, recent studies [2, 9] used BMI and WHRadjBMI SNPs to show that, in addition to BMI, body fat distribution (measured by WHRadjBMI) influences cardiovascular risk factors (including lipids, blood pressure, and diabetes), and is potentially more important than BMI in the development of subclinical atherosclerosis and stroke [2].

Recent MR analyses using GWAS data also indicate that insulin resistance (IR) and related measures may mediate the relationship between adiposity and cardiometabolic disease. One such effort generated a genetic proxy for IR based on meta-analysis of prior GWAS for triglycerides, high-density lipoprotein (HDL), and fasting insulin [10], and identified 53 associated SNPs. Moreover, a genetic instrument composed of those SNPs showed associations with risk of diabetes and cardiovascular disease (CVD), suggesting an IR-mediated relationship between adiposity and cardiometabolic disease. The genetic instrument was also associated with lower peripheral (that is, subcutaneous) adiposity, which could be interpreted as perturbed subcutaneous fat distribution playing a role in IR-related cardiometabolic disease. However, the choice to condition on BMI in one of the primary phenotypes (fasting glucose) may have induced an inverse relationship in the downstream analysis of IR-related SNPs with peripheral adiposity. Additional analyses, with and without conditioning on BMI, will be necessary to fully elucidate this relationship. Because these SNPs are drawn from summary data of GWAS based on three different traits (rather than from GWAS of an IR-defined trait), they may not provide a complete reflection of IR biology. Additionally, variants identified from such an analysis (combining three traits in a GWAS meta-analysis) may be pleiotropic; pleiotropic SNPs may affect multiple discrete pathways, potentially leading to biased estimates of disease risk [3]. Despite these caveats, creating a genetic proxy for IR represents an interesting approach for exploiting existing large-scale GWAS data to elucidate mediators of adiposity in cardiometabolic disease.

In addition to genetic variation, DNA methylation may also play an important role in the development of disease by modifying gene expression. A recent analysis of 5387 samples [11] sought to examine whether DNA methylation acts as a mediator between adiposity and cardiometabolic disease. After identifying 187 BMI-associated CpG sites (positions in the DNA where methylation may occur), the authors performed bidirectional MR, testing whether methylation changes cause BMI or vice versa. The results indicated that altered DNA methylation was a consequence—rather than a cause—of increased adiposity. Furthermore, the authors created genetic scores for a number of metabolic markers of BMI (blood pressure, hemoglobin A1c, HDL cholesterol, and insulin) and identified that these metabolic markers also influenced the 187 CpG sites (as opposed to methylation influencing the markers). These findings provide tantalizing evidence that, in addition to considering a standard etiologic framework (in which BMI increases systolic blood pressure, and higher systolic blood pressure increases risk of CVD), we should consider the possibility that BMI and other cardiometabolic traits may individually or collectively impact DNA methylation, thereby potentially causing disease through gene expression.

## Conclusions and implications for medicine

Collectively, genetic and genomic studies combined with MR have provided invaluable evidence that (1) general and central adiposity almost certainly have causal roles in the development of cardiometabolic disease; (2) a causal role for adiposity traits in the development of stroke subtypes is emerging [2]; and (3) potential mediators of adiposity in cardiometabolic disease, in addition to conventional risk factors like blood pressure, include insulin resistance, DNA methylation, and blood-based metabolites [8]. While these studies have yielded unique insights, challenges remain. For example, while GWAS have implicated genomic loci harboring risk variants, they cannot pinpoint the causal genes or mechanisms. Furthermore, GWAS only interrogate common variation, leaving rare variants essentially untested (and therefore under-represented in MR). Importantly, these limitations of GWAS do not necessarily hamper the applications of GWAS to MR. However, RCTs will, where feasible, almost always be necessary to establish robust evidence for the causality and efficacy of potential therapies, prior to the clinical implementation of findings from MR.

Despite these limitations, recent genetic and epigenetic findings have advanced our understanding of disease etiology and informed new research lines. Key questions remain, such as whether the BMI–methylation relationship represents a mechanism by which obese adults pass on harmful cardiometabolic risk to (lean) children, or whether the associations of BMI with multiple blood-based metabolites implicates markers that may represent potential drug targets. The challenge lies in addressing these questions, and translating the biological findings discussed here into effective therapies to combat obesity and the resulting health complications. As most drug targets are proteins, a natural extension is to investigate the associations of adiposity-related genetic risk scores with proteomics at scale. Aligning such findings to resources such as Open Targets (https://www.opentargets.org), a platform that integrates genomic data on genes and proteins with therapeutic relevance, may help to prioritize targets to take forward into clinical trials. Using genetics and epigenetics to identify therapies that can halt or ameliorate the mechanism by which adiposity leads to cardiometabolic disease will likely be more efficacious than (or at the very least enhance) current conventional advice to address adiposity through improved diet or physical activity, advice that has had minimal impact on deleterious global adiposity trends and its consequences.

In summary, genetic and epigenetic studies have contributed to our understanding of the role of adiposity in cardiometabolic disease and illuminated potential mechanisms. Although the field has yet to find a pivotal drug target from genetic studies, as has occurred for the *PCSK9* gene in CVD treatment, insights from recent efforts provide promising paths forward that could result in substantial public health gains for global communities increasingly affected by obesity and its sequelae.

## Abbreviations

BMI: Body mass index
CHD: Coronary heart disease
CVD: Cardiovascular disease
GWAS: Genome-wide association study
HDL: High-density lipoprotein
IR: Insulin resistance
MR: Mendelian randomization
SNP: Single nucleotide polymorphism
WHR: Waist-to-hip ratio
WHRadjBMI: Waist-to-hip ratio, adjusted for BMI
